# Small noncoding RNA sRNA0426 is involved in regulating biofilm formation in *Streptococcus mutans*

**DOI:** 10.1002/mbo3.1096

**Published:** 2020-07-06

**Authors:** Luoping Yin, Wenhui Zhu, Dongru Chen, Yan Zhou, Huancai Lin

**Affiliations:** ^1^ Guanghua School of Stomatology Hospital of Stomatology Sun Yat‐Sen University Guangzhou China; ^2^ Guangdong Provincial Key Laboratory of Stomatology Guangzhou China

**Keywords:** biofilm formation, dental caries, exopolysaccharides, small RNAs, *Streptococcus mutans*

## Abstract

Evidence suggests that small noncoding RNAs (sRNAs) are involved in the complex regulatory networks governing biofilm formation. Few studies have investigated the role of sRNAs in *Streptococcus mutans* (*S*.* mutans*). In the present study, the association between sRNA and biofilm formation in *S*.* mutans* was explored. sRNAs that are differentially expressed in the biofilm and planktonic states of this bacterium were identified by quantitative real‐time PCR (qRT‐PCR). Confocal laser scanning microscopy was used to investigate the characteristics of biofilm formation in a standard strain of *S*.* mutans* (UA159, ATCC 700610) and ten clinical strains. Bioinformatics analyses were employed to predict and examine potential sRNA regulatory pathways. The results showed that sRNA0426 has a strong positive relationship with dynamic biofilm formation. Moreover, sRNA0426 expression was positively correlated with exopolysaccharide (EPS) production. Bioinformatics analyses showed that sRNA0426 is involved in biofilm formation such as metabolic pathways, especially carbon metabolism. Five target mRNAs (GtfB, GtfC, GtfD, ComE, and CcpA) involved in the synthesis of EPS were selected for further evaluation; the expression levels of three of these mRNAs (GtfB, GtfC, and CcpA) were positively correlated with sRNA0426 expression levels, and the expression level of one (ComE) was negatively correlated. In conclusion, the results suggested that sRNA0426 may play an important and positive role in the biofilm formation of *S*.* mutans* and provide novel insight into the *S*.* mutans* biofilm regulatory network.

## Graphical abstract

This study focuses on the role of small noncoding RNA both in standard *Streptococcus mutans* and clinical strain biofilms. sRNA0426 is potentially involved in the biofilm formation of *S. mutans*. The results of this study provide new insights into the complex biofilm regulatory network in *S. mutans*, which infers a new possibility for dental caries prevention.

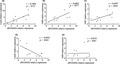

## 1 INTRODUCTION

*Streptococcus mutans* (*S*.* mutans*), the bacterium currently recognized as the main microbiological cause of dental caries, depends on the formation of biofilms to exert its virulence (Klein, Hwang, Santos, Campanella, & Koo, 2015). Compared to the planktonic form, biofilm formation provides *S*.* mutans* with a better opportunity to adapt to the changing environment in the oral cavity over a planktonic condition (Flemming & Wingender, 2010; Krzysciak, Jurczak, Koscielniak, Bystrowska, & Skalniak, 2014; Welin‐Neilands & Svensater, 2007). Therefore, it is important to explore the mechanism of biofilm formation in *S*.* mutans*.

Small noncoding RNAs (sRNAs) are typically 50–400 nt in length and continuously fine‐tune regulatory networks to enable concentration‐specific responses to environmental cues by sequestering, antagonizing, or activating regulatory mRNAs and proteins (Chambers & Sauer, 2013). sRNAs play pivotal roles in regulating gene expression under various conditions, thereby promoting adaptation to a changing environment, especially the biofilm microenvironment (Faizan et al., 2017; Roop et al., 2017; Tsai et al., 2013). It is increasingly appreciated that sRNAs are involved in the complex regulatory mechanisms that govern biofilm development, including the switch between planktonic and biofilm states in bacteria (Caldelari, Chao, Romby, & Vogel, 2013; Chambers & Sauer, 2013; Ghaz‐Jahanian, Khodaparastan, Berenjian, & Jafarizadeh‐Malmiri, 2013). For example, Zhao, Koestler, Waters, and Hammer (2013) found that Qrr sRNAs simultaneously negatively and positively regulate expression of the hapR gene and the vca0939 gene, respectively, to promote biofilm formation in *Vibrio cholera*. In *Streptococcus sanguinis*, two sRNAs that negatively regulate biofilm formation by inhibiting the expression of the target pilT gene were recently identified (Ota et al., 2018).

Additionally, Lee and Hong (2012) revealed more than 900 sRNAs and highlighted the importance of sRNAs in *S*.* mutans*. In a previous study, we established a library of 736 differentially expressed candidate sRNAs associated with initial adhesion in *S*.* mutans* UA159 by RNA deep sequencing (Zhu, Liu, Liu, Zhou, & Lin, 2018). Moreover, we observed a consistent correlation between the expression of sRNAs and initial adhesion ability in 100 clinical strains of *S*.* mutans* (Zhu et al., 2018). Initial adhesion is the first step in biofilm formation, the processes of which include reversible attachment, irreversible attachment, maturation, and dispersion (Hinsa, Espinosa‐Urgel, Ramos, & O'Toole, 2003). The microbial composition and structure change dynamically during biofilm formation. Although sRNAs are widely considered to act as key regulators in biofilm formation (Svenningsen, 2018), there has been limited investigation of the role of these molecules in the dynamic process of biofilm formation (Kreth, Liu, Chen, & Merritt, 2015), and it remains unknown whether sRNA exerts an important role during the process of biofilm formation in *S*.* mutans*.

In this study, we first screened sRNAs associated with biofilm formation in the standard strain of *S*.* mutans* UA159 and then investigated the potential association between sRNAs and biofilm formation and the production of exopolysaccharide (EPS) in clinical strains of *S*.* mutans*. Bioinformatics analysis was used to predict and verify the potential regulatory mechanisms employed by candidate sRNAs. The results highlight the function of sRNAs in the dynamic regulation of biofilm formation and provide a promising avenue for developing novel methods of caries prevention by targeting *S*.* mutans*.

## 2 METHODS

### 2.1 Bacterial strain and culture conditions

The strains used in the present study included the standard strain of *S*.* mutans* (UA159ATCC 700610) and clinical isolates. Clinical isolates were obtained from an epidemiological survey conducted in Guangdong Province, People's Republic of China, in 2015 (Yu et al., 2015). The survey was conducted among 5‐year‐old children. A total of 215 clinical strains were isolated from 215 children with different caries status (Zhu et al., 2018). From these isolates, 10 clinical strains were randomly selected. The *S*.* mutans* strains were grown in brain heart infusion (BHI) broth (Oxoid) overnight under anaerobic conditions (80% N_2_, 20% CO_2_) at 37°C. The optical density at 600 nm (OD_600_) of overnight‐cultured strains was measured using a microplate reader (Bio‐tek, Epoch 2, America). UA159 suspensions (OD_600_ = 0.7) were inoculated at 1:20 into fresh BHI in round‐bottom 6‐well plates to obtain planktonic cells; the same suspensions (OD_600_ = 0.7) were inoculated at 1:20 into fresh BHI in flat‐bottom 6‐well plates and incubated for 4 h, 6 h, 12 h, and 24 h to monitor the dynamic biofilm formation process of *S*.* mutans*.

### 2.2 RNA extraction

Planktonic bacteria were collected by centrifugation at (13201 *g*) for 5 min. Biofilm bacteria were scraped from plates and centrifuged at (13201 *g*) for 5 min. Total RNA extraction was performed according to the method described by Zhu et al. (2017). Briefly, total RNA was stabilized with RNAprotect Bacteria Reagent (Qiagen) before extraction. Biofilms were harvested and washed three times with phosphate‐buffered saline (PBS). The total RNA of biofilm cells was purified using a miRNeasy Mini Kit (Qiagen). A Thermo Scientific NanoDrop 2000 instrument (NanoDrop Technologies) and an Agilent 2100 system (Agilent Technologies) were used to assess RNA quality and quantity.

### 2.3 Quantitative real‐time PCR (qRT‐PCR)

The top twenty significantly differentially expressed sRNAs were selected as candidates from our sRNA library established in a previous study (Zhu et al., 2018). These candidate sRNAs were further analyzed between planktonic and biofilm conditions of *S*.* mutans* at 24 h by qRT‐PCR. cDNA was synthesized using a Mir‐X miRNA First‐Strand Synthesis Kit (Takara and Clontech) according to the manufacturer's recommended protocol. qRT‐PCR was performed using a LightCycler 96 Real‐Time System and the SYBR Premix Ex Taq II Kit (Takara and Clontech). The primers used for sRNAs in this study are listed in Table 1. The reaction conditions were 95°C for 30 s followed by 40 cycles of 95°C for 15 s and 60–63°C for 30 s. The expressions of sRNAs were normalized to the 16S rRNA expression level.

**TABLE 1:** Primers for candidate sRNAs and 16S rRNA

sRNA ID	Primers	
	Forward	Reverse
sRNA0698 (Zhu et al., 2018)	CTATTTCTGTTCTATTTTACCACA	Universal primer
sRNA0593 (Zhu et al., 2018)	CGCCAATCATTTCATTTTCCA	CCTACGTTTCCCGTGCCTAA
sRNA0074*	TACTGGAATAATGTTTAATTTTACT	Universal primer
sRNA0522*	CAATAGTAATAAGGTAAAGTGCG	GTATCTCGTAAATACTACAAAGAATT
sRNA0426*	ATTGGATAAGACCGTTACACA	AAATAGCGAGACAAGAAAGTT
sRNA0413*	AATAATAAGTCCGCAAAAATC	AAGGTGGATTAGGTAAAGATG
sRNA0650*	TTAGCATCTTTTACATCACAATA	TGATTCTTCTTTATGGGACA
sRNA0146*	AGCTAGTTGCTATAATTAATAATTT	TTCTCTTCAGTTAGACAATCTCT
sRNA0215*	TTGTGAAGCTCTCAATAAGTT	GATGTATCCAATGAATCAGTGA
sRNA0120*	TAAGCGTAAGCGGCAAAACT	AATAGCTGGGCTTCAGGTGC
sRNA0118*	AATATTGATTTTGACCTGCAT	GATTTTAGGCTAACTTTTGAGAT
sRNA0379*	AGTGCTTCTTCAATTTTATCCATC	GGCAAGGATAGAATGGTTGT
sRNA0250*	GCCATTTAAGATTCGGACTA	AGGAAGTGAATAAGTATGAAAGT
sRNA0301*	CTAAAGGGCAATAAAATATGTGA	GAAGCGTTTCCTATAAATTCTAT
sRNA0600*	TGTATTTGTTTCGGACCTTA	CGCTATTACGCGATATTCT
sRNA0656 (Zhu et al., 2018)	TATGGGGGATAAGATATGCTATGAT	Universal primer
sRNA0330 (Zhu et al., 2018)	TTTATTAGAAAGGAACAGTTTTG	Universal primer
sRNA0187 (Zhu et al., 2018)	CGTTCCGTCAAATAACCAAAGTG	AAGGAGAATGGTAATTCCGCTTT
sRNA0329 (Zhu et al., 2018)	GCAAAACTGTTCCTTTCTAATAA	Universal primer
sRNA0679 (Zhu et al., 2018)	AATCTCAAGCAAAGACTTTTTAGA	Universal primer
16S rRNA	CTTACCAGGTCTTGACATCCCG	ACCCAACATCTCACGACACGAG

* The primers were designed by the technical staff from the TakaRa company. The universal primer was commercially supplied with the Mir‐X miRNA qRT‐PCR SYBR kits (TaKaRa).

The most highly expressed sRNA associated with biofilm formation from the analyzed 20 sRNAs was selected for further analysis. Five target mRNAs of the candidate sRNA were selected, and their possible functional roles were preliminarily explored. The primers used in the qRT‐PCR analysis of the mRNAs are listed in Table 2. Synthesis of cDNA and qRT‐PCR were performed as described above. The expression level of each gene was determined in triplicate. Expression levels were calculated using the ^2−ΔΔ^Ct method (Livak & Schmittgen, 2001).

**TABLE 2:** Primers for potential target mRNAs

Gene ID	Primers	
	Forward	Reverse
*ComE* (Hung et al., 2011)	AGCCCATAAGCTCTGCCTTT	AGCGATGGCACTGAAAAAGT
*CcpA* (Wen & Burne, 2002)	ATTGACCGTCTTGATTATC	AGCATTAGCAATATTAGGG
*Gtf*B (Gao et al., 2018)	AGCAATGCAGCCAATCTACAAAT	ACGAACTTTGCCGTTATTGTCA
*GtfC* (Gao et al., 2018)	CTCAACCAACCGCCACTGTT	GGTTTAACGTCAAAATTAGCTGTATTAGC
*GftD* (Gao et al., 2018)	ACAGCAGACAGCAGCCAAGA	ACTGGGTTTGCTGCGTTTG

### 2.4 Crystal violet (CV) staining assay

The CV staining assay was used to evaluate the biofilm biomass of *S*.* mutans* (Weerasekera et al., 2016). *Streptococcus mutans* UA159 and the 10 clinical strains were incubated in flat‐bottom 96‐well plates under anaerobic conditions for 4 h, 6 h, 12 h, and 24 h. Then the contents of the 96‐well plates were then removed, and the plates were washed three times with phosphate‐buffered saline (PBS) to remove nonadherent cells. The washed biofilms were fixed with 95% methanol for 15 min and washed again. The biofilms were stained with 0.1% (wt/vol) CV solution for 15 min at room temperature. After thorough removal of the excess liquid, the remaining CV was dissolved in 200 μl of 95% ethanol for 15 min, and 100 μl of the sample was transferred to a new plate for OD_600_ measurement.

### 2.5 Confocal laser scanning microscopy (CLSM)

For analysis of EPS production, 1 μM Alexa Fluor 647 (Invitrogen) and 2.5 μM SYTO 9 (Invitrogen) were used to label dextran and bacterial cells, respectively (Huang et al., 2017). COMSTAT was used to analyze the biomass of EPS (μm^3^/μm^2^). The three‐dimensional architecture of the biofilms was reconstructed using Imaris 8.0.2 (Bitplane). Three independent experiments were performed for each condition, and images of five random fields were collected for each sample.

### 2.6 Bioinformatics analysis of candidate sRNAs

We predicted the structures of candidate sRNA using RNAfold (http://rna.tbi.univie.ac.at/cgi‐bin/RNAWebSuite/RNAfold.cgi). According to sequence data for *S*.* mutans* UA159 (AE014133.2), functional annotation of sRNA was performed with the Kyoto Encyclopedia of Genes and Genomes analyses (KEGG) and Database for Annotation, Visualization and Integrated Discovery (DAVID) software (http://david.abcc.ncifcrf.gov/). The binding sites of sRNAs in putative target mRNAs were predicted by intaRNA (http://rna.informatik.uni‐freiburg.de/IntaRNA/Input.jsp).

### 2.7 Statistical analyses

Each experiment was independently repeated three times. GraphPad Prism version 7.0a (GraphPad Software, San Diego, CA, USA) and IBM SPSS 24.0 (IBM, Armonk, NY, USA) were used to analyze the data. The means and standard deviations of all continuous variables were computed. The data were assessed for normal distribution and sphericity; an unpaired *t* test was used for two conditions, and repeated measures analysis of variance was used for multiple time points (*p* < 0.05). The Spearman rank correlation coefficient was applied with a *p*‐value of <0.05 for correlation testing.

## 3 RESULTS

### 3.1 Screening for the most highly differentially expressed sRNAs associated with biofilm formation

To obtain the most relevant sRNAs associated with biofilm formation, we screened the top 20 differentially expressed sRNAs from our previous study in the standard strain of *S*.* mutans* (UA159, ATCC 700610). Among the 20 sRNAs, 18 were successfully detected with 14 were upregulated in cultures with biofilm status relative to those with planktonic status, and 4 were downregulated. Two sRNAs (sRNA0250 and sRNA0656) expressed so unstably after multiple repeated studies that the detection of these two sRNAs was not shown in Table 3. sRNA0426 was the most highly differentially expressed sRNA. Its expression was 5.87 times higher in the biofilm state than in the planktonic state (*p* < 0.001, Table 3).

**TABLE 3:** Analysis of the differential expression of 20 sRNAs between planktonic and biofilm conditions in standard *Streptococcus mutans* at 24 h

sRNAs	log_2_ Fold change (Biofilm/Planktonic)		Fold change (Biofilm/Planktonic)	*t* value	*p*‐value
sRNA0426	2.55	5.87		26.09	<0.001
sRNA0379	2.33	5.01		54.82	<0.001
sRNA0650	2.19	4.56		54.31	<0.001
sRNA0413	2.13	4.38		71.42	<0.001
sRNA0600	1.99	3.97		19.11	<0.001
sRNA0522	1.79	3.46		55.26	<0.001
sRNA0698	1.29	2.45		20.36	<0.001
sRNA0593	1.27	2.42		9.86	<0.001
sRNA0215	1.14	2.20		13.78	<0.001
sRNA0120	0.84	1.79		7.52	<0.001
sRNA0146	0.81	1.75		8.99	<0.001
sRNA0118	0.69	1.62		19.42	<0.001
sRNA0301	0.54	1.45		17.93	<0.001
sRNA0074	0.17	1.13		2.74	0.021
sRNA0329	−0.66	0.63		−7.43	<0.001
sRNA0187	−1.18	0.44		−13.44	<0.001
sRNA0330	−1.23	0.43		−12.83	<0.001
sRNA0679	−1.25	0.42		−15.81	<0.001
sRNA0250	‐	‐		‐	‐
sRNA0656	‐	‐		‐	‐

Note

Expression of the 20 selected sRNAs under standard *Streptococcus mutans* biofilm conditions compared with planktonic conditions at 24 h. 18 sRNAs were differentially expressed between the two conditions; of these, sRNA0426 was the most significantly upregulated sRNA. The expression of sRNA0250 and sRNA0656 under these two conditions was not measured stably. The sequencing data for these sRNAs were obtained from (Zhu et al., 2018).

### 3.2 Expression of sRNA0426 during biofilm formation in standard and clinical strains of *S. mutans*

**FIGURE 1:**
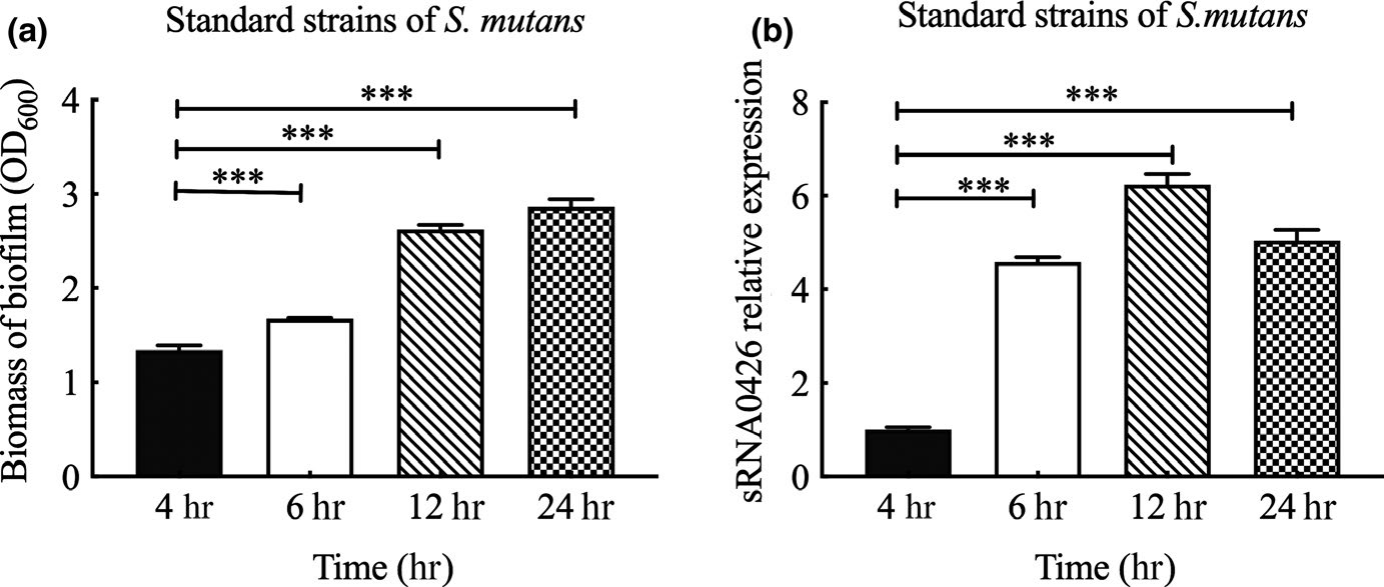
Characteristics of biofilm formation and expression of sRNA0426 in the standard strain of *Streptococcus mutans*. (a) The biomass of biofilm (OD_600_) during biofilm formation by the standard strain was evaluated using the CV assay. (b) Dynamic expression analysis of sRNA0426 in the standard strain was performed. The level of expression of sRNAs at 4 h was defined as 1.0. Data represent the mean ± *SD*. *p < 0.05, **p < 0.01, ***p < 0.001

**FIGURE 2:**
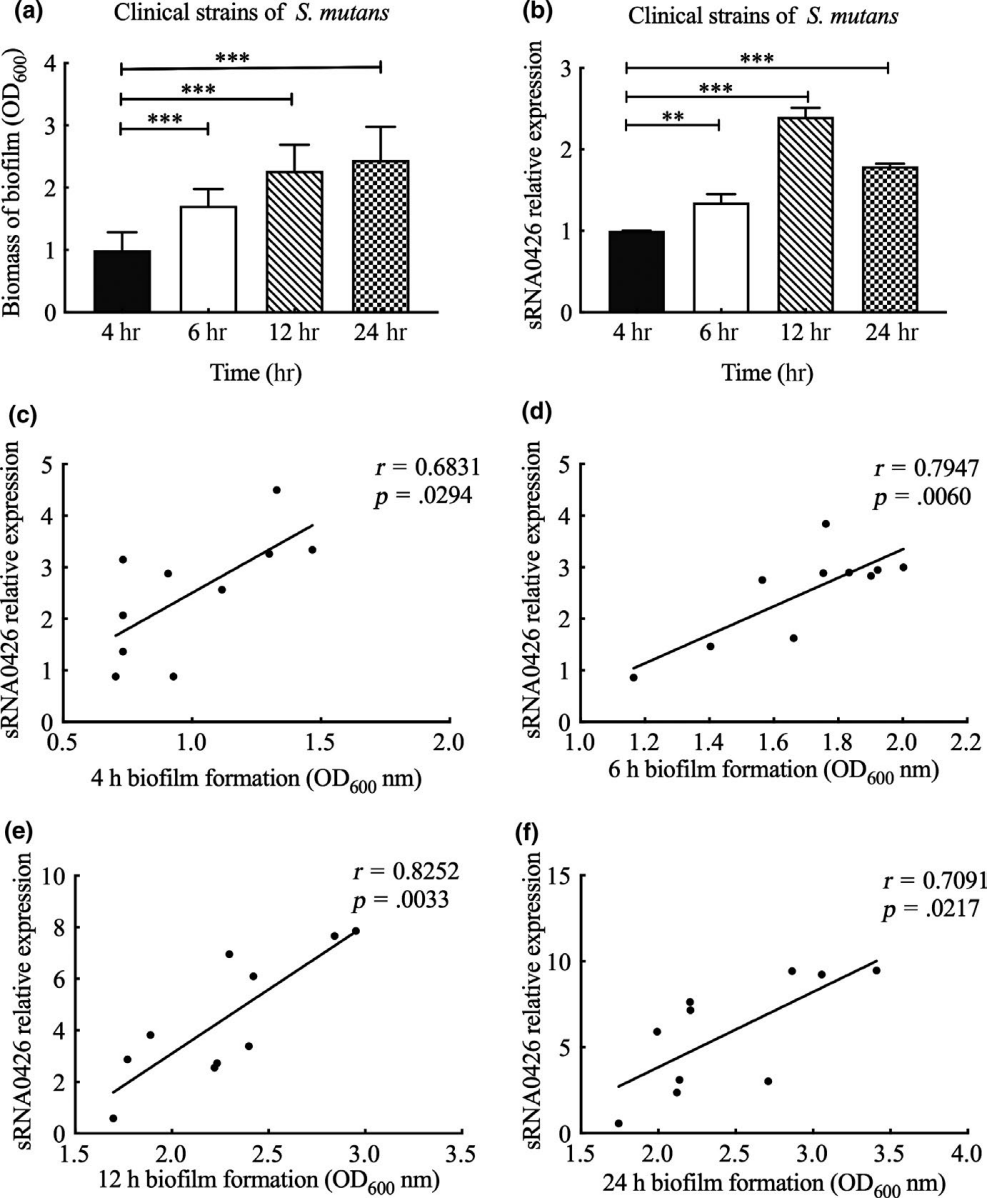
Characteristics and association of biofilm formation with the expression of sRNA0426 in clinical strains of *Streptococcus mutans*. (a) Biofilm biomass (OD_600_) during biofilm formation by clinical strains of *S*.* mutans*. (b) Dynamic expression analysis of sRNA0426 in clinical strains of *S*.* mutans*. The level of expression of sRNAs at 4 h was defined as 1.0. (c‐f) The level of expression of sRNA0426 in strain 5521 was defined as 1.0. Spearman correlation analysis of sRNA0426 expression with biofilm formation is shown in the figure for the 10 clinical isolates at 4 h, 6 h, 12 h, and 24 h. Data represent the mean ± *SD*. *p < 0.05, **p < 0.01, ***p < 0.001

To further verify the relationship between sRNA0426 and biofilm formation, we first evaluated the biofilm biomass by CV assays and measured the expression of sRNA0426 at 4 h, 6 h, 12 h, and 24 h in the standard strain of *S*.* mutans*. The biofilm biomass increased from 4 h to 24 h during the biofilm formation process in the standard strain (*p* < 0.05, Figure 1a). Also, expression of sRNA0426 changed dynamically during biofilm formation in the standard strain, gradually increasing from 4 h to 12 h and then decreasing slightly at 24 h, with a peak at 12 h (*p* < 0.001) (Figure 1b). We observed a similar trend in the clinical strains of *S*.* mutans* (Figure 2a,b). There was a positive correlation between sRNA0426 expression and biofilm biomass in the clinical strains at various times (4 h, 6 h, 12 h, and 24 h). From 4 h to 12 h, the correlation strengthened as the biofilm formation capability of the 10 clinical strains increased, although the correlation weakened from 12 h to 24 h (Figure 2c‐f). The strongest correlation between sRNA0426 expression and biofilm formation capability was observed at 12 h (*r* = 0.8252, *p* = 0.0033) (Figure 2e).

### 3.3 Relationship between expression of sRNA0426 and EPS

**FIGURE 3:**
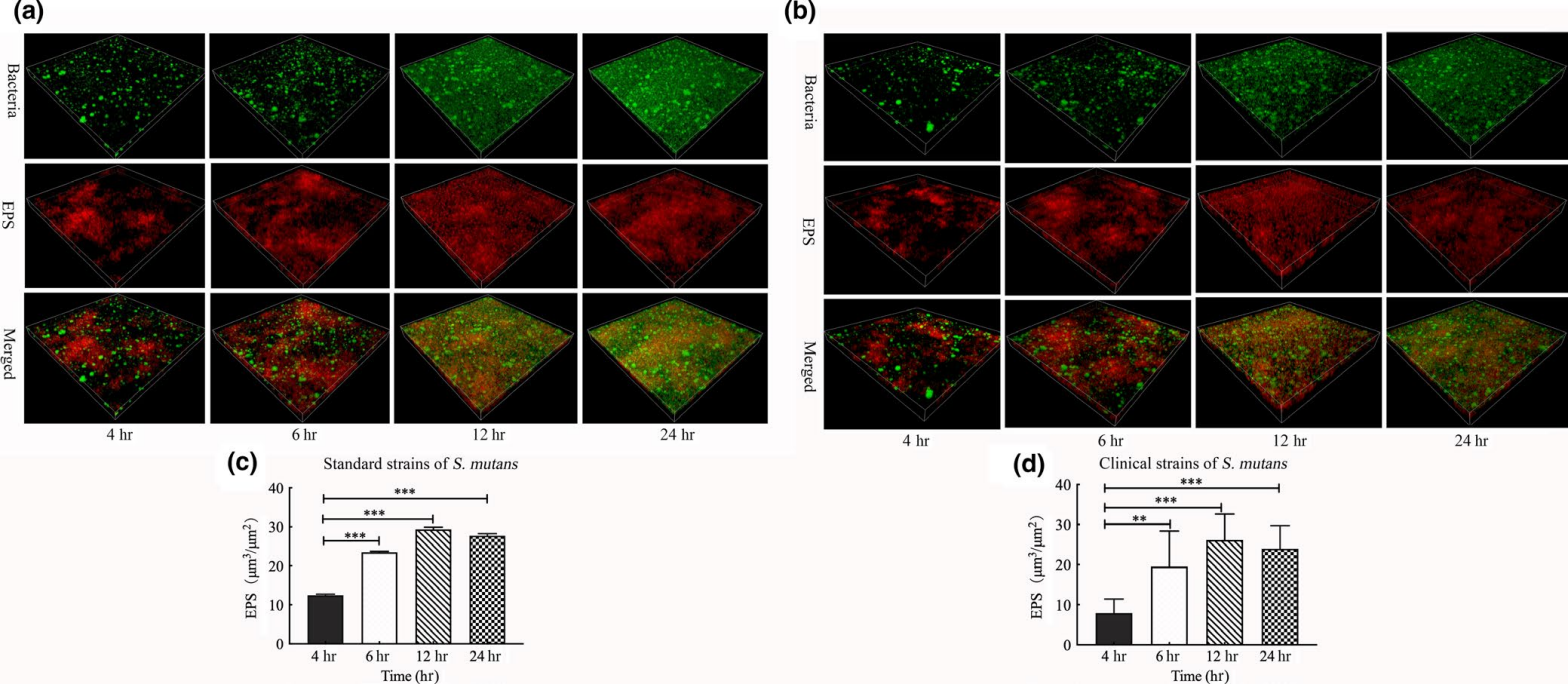
EPS analysis of *Streptococcus mutans*. (a‐b) Three‐dimensional reconstructions of live bacteria and EPS in biofilms of standard *S*.* mutans* and one representative clinical strain at 4 h, 6 h, 12 h, and 24 h. EPS is labeled in red (Alexa Fluor 647), and bacterial cells are labeled in green (SYTO9). (c‐d) EPS biomasses of standard *S*.* mutans* and 10 *S*.* mutans* clinical strains at 4 h, 6 h, 12 h, and 24 h. EPS biomasses were calculated according to 5 random sites in each CLSM micrograph image. Each determination was repeated three times

**FIGURE 4:**
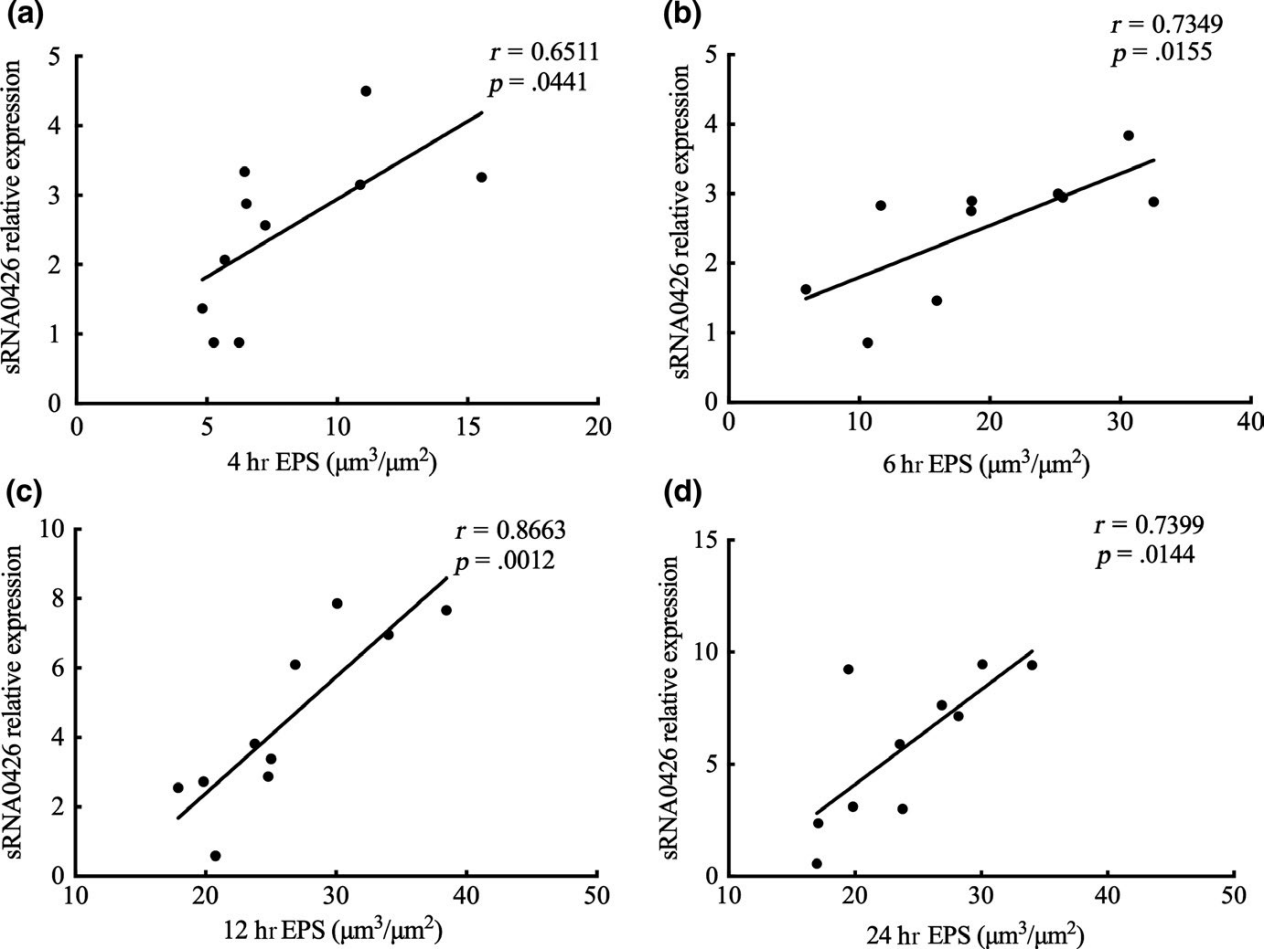
Correlation of sRNA0426 expression with EPS. (a‐d) The level of expression of sRNA0426 in strain 5521 was defined as 1.0. Spearman correlation analysis of sRNA0426 relative expression with EPS is shown in the figure for the 10 clinical isolates at 4 h, 6 h, 12 h, and 24 h. *p < 0.05, **p < 0.01, ***p < 0.001

EPS forms the core of the matrix scaffold and provides binding sites that promote the accumulation of microorganisms on the tooth surface and the establishment of pathogenic biofilms (Bowen, Burne, Wu, & Koo, 2018). Thus, to further explore the association between sRNA0426 and EPS, we specifically analyzed EPS by CLSM. According to the confocal micrographs of EPS, the bacteria became increasingly encased or surrounded by EPS with time, but no change was apparent from 12 h to 24 h (Figure 3a,b). The highest biovolume of EPS was observed at 12 h in the biofilm of both the standard and clinical strains of *S*.* mutans* (*p* < 0.05) (Figure 3c,d), and the trend of the change in the amount of EPS was consistent with the dynamic expression of sRNA0426 during biofilm formation. We then analyzed the relationship between expression of sRNA0426 and the amount of EPS in the biofilms of the 10 clinical strains at 4 h, 6 h, 12 h, and 24 h of culture. The results obtained with the clinical strains suggest that the expression level of sRNA0426 correlates positively with the amount of EPS present during biofilm formation. The strongest correlation was observed at 12 h (*r* = 0.8663, *p* = 0.0012) (Figure 4c). These results indicate that sRNA0426 may play a positive role in the production of EPS in *S*.* mutans* biofilms.

### 3.4 Functional annotation of sRNA0426 using bioinformatics analyses

**FIGURE 5:**
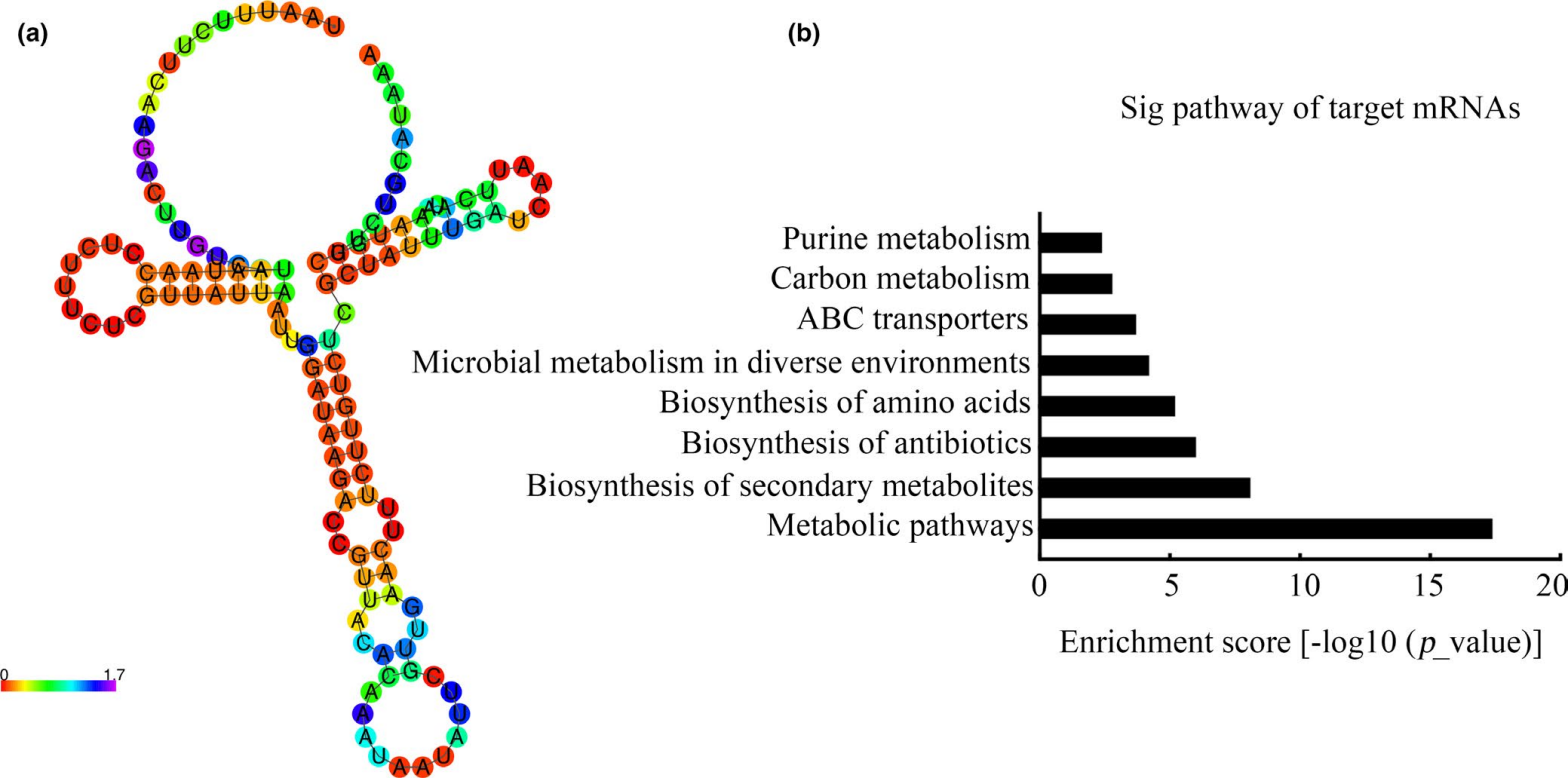
Bioinformatics analyses of sRNA0426. (a) The secondary structure of sRNA0426 predicted by RNAfold. Different colors indicate the probabilities of base composition in the secondary structure as graphic symbols. sRNA0426 possesses a stem‐loop structure with a dG value of −18.7 kcal/mol. (b) Biological pathways predicted by KEGG analysis for target mRNAs of sRNA0426 at *p* < 0.05

Considering the importance of secondary structures in stabilizing sRNAs, the secondary structure of sRNA0426 was predicted using RNAfold. It is reported that sRNA0426 possesses a stem‐loop structure with a Δ*G* value of −18.7 kcal/mol (Figure 5a). To the best of our knowledge, sRNA0426 is located on the antisense mRNA strand between SMU_1238c and SMU_1239 (Table A1). To explore the potential mechanism by which sRNA0426 regulates *S*.* mutans* biofilm formation, KEGG pathway annotation was used to investigate the sRNA0426 regulatory pathway, revealing eight pathways that are significantly regulated by sRNA0426 (*p* < 0.05) (Figure 5b). Specifically, most of the pathways are involved in biofilm formation, such as metabolic pathways, especially carbon metabolism. The results of the KEGG analysis of biological pathways for the other sRNAs are presented in Appendix B (https://doi.org/10.6084/m9.figshare.12310133). The KEGG analysis for the other differential sRNAs expression showed a potential similar pathway with sRNA0426 and that there might be several sRNAs involved in the biofilm regulatory network in *S*.* mutans*.

**FIGURE 6:**
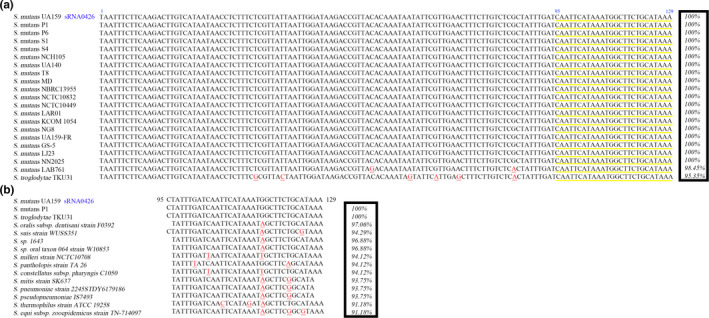
(a) Sequence alignment of putative homologs of confirmed sRNA0426 in *Streptococcus mutans*. (b) Sequence analysis of the seed sequence of sRNA0426. Only the representative 14 strains of these species were listed in the figure

To determine whether similar putative sRNAs are present in other bacteria, we searched for sequences homologous of sRNA0426 using BLASTN. The results are shown in Figure 6. A sequence was only considered to be conserved when the coverage between the query and subject sequences was higher than 75% and the nucleotide identity was higher than 65% (*E*‐value = 10^−5^, word = 11). The results suggest that sRNA0426 might be conserved in *Streptococcus* species, primarily in *S*.* mutans* strains and *Streptococcus troglodytae* (*S*.* troglodytae*). The genomes of 14 *S*.* mutans* strains were found to cover 100% of the sequence of sRNA0426. The *S*.* mutans* strain LAB761 and *S*.* troglodytae* separately cover 98.45% and 95.35% of the sequence respectively (Figure 6a). Furthermore, 14 *streptococcus* species including 105 strains have a 24%–27% query cover of sRNA0426. The BLASTN results of the representative 14 *streptococcus* species were shown in Figure 6b, and more details about the total 105 strains were presented in Appendix C (https://doi.org/10.6084/m9.figshare.12310133). We consider it specific seed sequences for the function of sRNA0426 in *S*.* mutants*.

### 3.5 Relationship of sRNA0426 and potential target mRNAs

**FIGURE 7:**
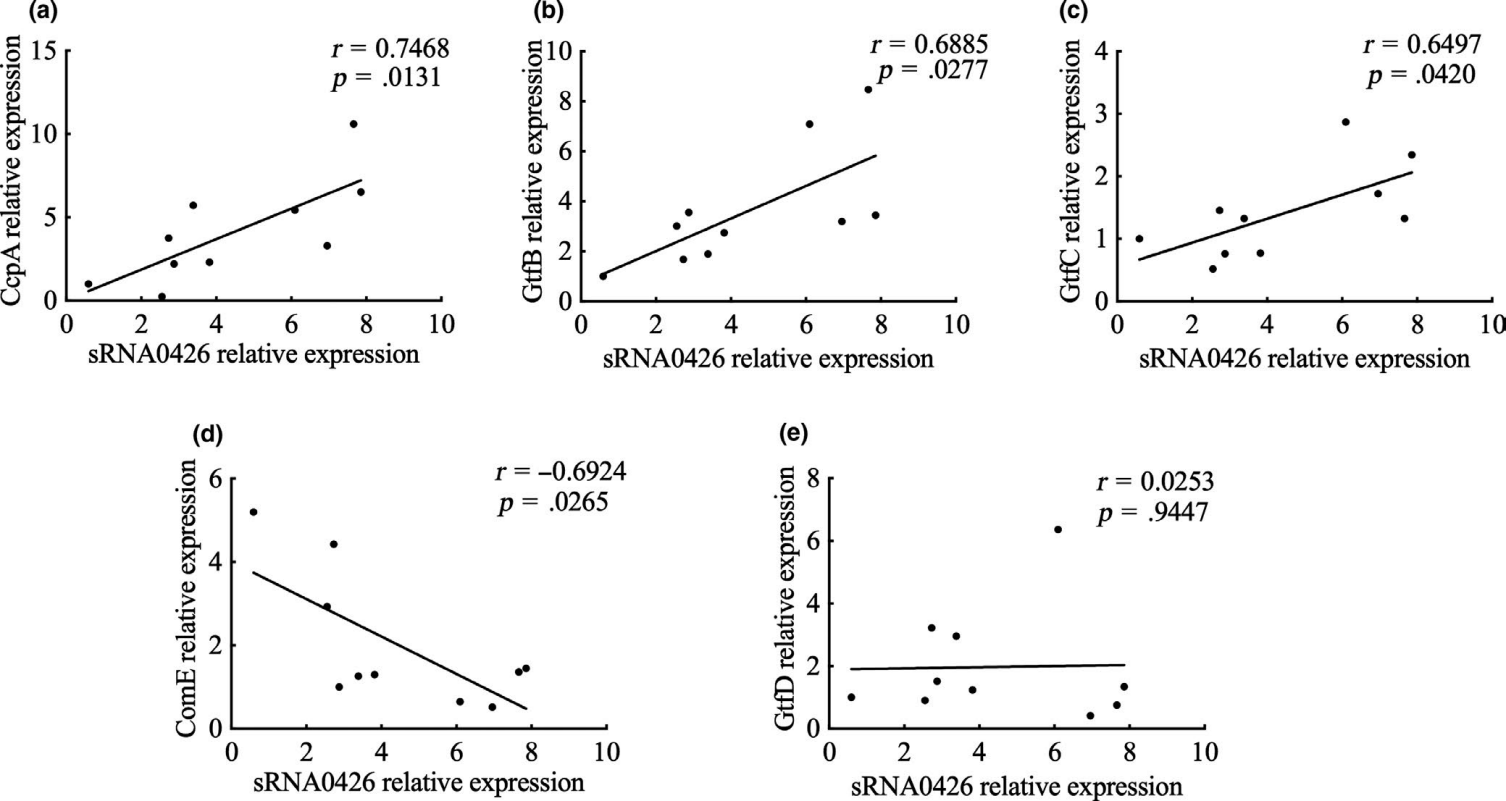
The potential role of sRNA0426 in biofilm formation. (a‐e) The level of expression of sRNA0426 and potential target mRNAs in strain 5521 was defined as 1.0. Spearman correlation analysis of sRNA0426 expression with ComE, GtfBCD, and CcpA is shown in the figure for the 10 clinical isolates at 12 h

**FIGURE A1:**
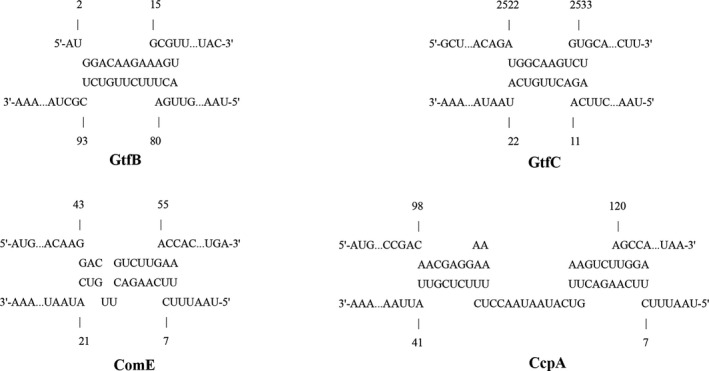
Putative binding sites for sRNA0426 in GtfBC, ComE, and CcpA as predicted by intaRNA. The bases of sRNA0426 are shown on the bottom

To further explore the function of sRNA0426, we examined the association between sRNA0426 and five potential target mRNAs predicted by bioinformatics (GtfB, GtfC, GtfD, ComE, and CcpA) at 12 h, when the strongest correlation was observed between sRNA0426 and biofilm biomass together with EPS. According to the results, sRNA0426 expression showed a significantly positive relationship with GtfB, GtfC, ComE, and CcpA expression (*p* < 0.05) but no significant relationship with GtfD expression (Figure 7). Potential binding sites were also predicted by intaRNA (Figure A1). The presence of binding sites between potential target mRNAs and sRNA0426 provides evidence for a regulatory role of sRNA0426.

## 4 DISCUSSION

Biofilm formation of *S*.* mutans* is a dynamic process that involves biofilm‐specific genetic mechanisms and regulatory networks that allow the bacterium to adapt to a changing microenvironment (Krzysciak et al., 2014). sRNAs are reported to exert broad regulation by directly targeting a large number of mRNAs, thereby playing a crucial role in biofilm formation (Caldelari et al., 2013; Chambers & Sauer, 2013). However, identification and further analysis of biofilm‐associated sRNAs in *S*.* mutans* have yet to be performed, especially in clinical strains. In this study, we detected the expression of sRNAs associated with biofilm formation and preliminarily investigated the potential function of sRNAs during biofilm formation both in standard *S*.* mutans* strain and clinical strains. Genes that are differentially expressed between biofilm and planktonic states are considered to be highly associated with biofilm formation. In *Acinetobacter baumannii*, Alvarez‐Fraga et al. (2017) found that sRNA13573 was expressed more highly in biofilms than during planktonic states and verified that sRNA13573 was involved in biofilm formation. A previous study also showed that biofilm‐associated genes exhibit different expression profiles in *S*.* mutans* under biofilm and planktonic conditions (Shemesh, Tam, & Steinberg, 2007). In our study, the expression of sRNA0426 was significantly higher in biofilms than in the planktonic state, and it changed dramatically during the biofilm formation process, showing a strong association with biofilm formation. Together with the correlation between sRNA0426 and biofilm biomass, the data suggest that sRNA0426 is associated with biofilm formation in *S*.* mutans*.

Biofilms are highly dynamic and structured communities of bacteria enmeshed in a self‐produced matrix of extracellular polymeric substances (Flemming & Wingender, 2010; Flemming et al., 2016). EPS forms the core of the matrix scaffold and provides a binding site for bacterial cells, mediating their adherence to form mature biofilms (Koo, Falsetta, & Klein, 2013). As attractive and effective regulators, sRNAs have an important function in the production of EPS. Liu reported that the sRNA HmsB (sR035) promotes biofilm formation by increasing EPS production and that HmsA (sR084) activates biofilm formation by modulating the intracellular level of c‐di‐GMP molecules to determine EPS production in *Yersinia pestis* (Liu et al., 2016). Additionally, sRNAs cooperate with Hfq to regulate EPS production in *Erwinia amylovora* (Zeng, McNally, & Sundin, 2013). In the present study of *S*.* mutans*, sRNA0426 displayed a positive correlation with EPS. The results suggest that sRNA0426 plays an important role in *S*.* mutans* biofilm formation through the production of EPS.

Synthesis of EPS is determined by carbon metabolism, which in *S*.* mutans* is mainly controlled by glucosyltransferases (*gtfs*). *GtfBC* metabolizes sucrose to produce water‐insoluble glucans, and *gtfD* synthesizes predominantly soluble glucans to establish the EPS matrix (Li & Burne, 2001). The activities of *gtfs* are controlled by regulators. For example, *comE* is part of two‐component signal transduction systems and it is an occluded RNA polymerase that binds to the coding region of *gtfC* to abort its expression, thereby interfering with carbon metabolism and biofilm formation (Hung et al., 2011). Furthermore, *ccpA* plays a critical role in the response to carbon source availability by affecting the stability of biofilms in *S*.* mutans*, and the *gtfBC* genes require *ccpA* for optimal expression (Wen & Burne, 2002).

**FIGURE A2:**
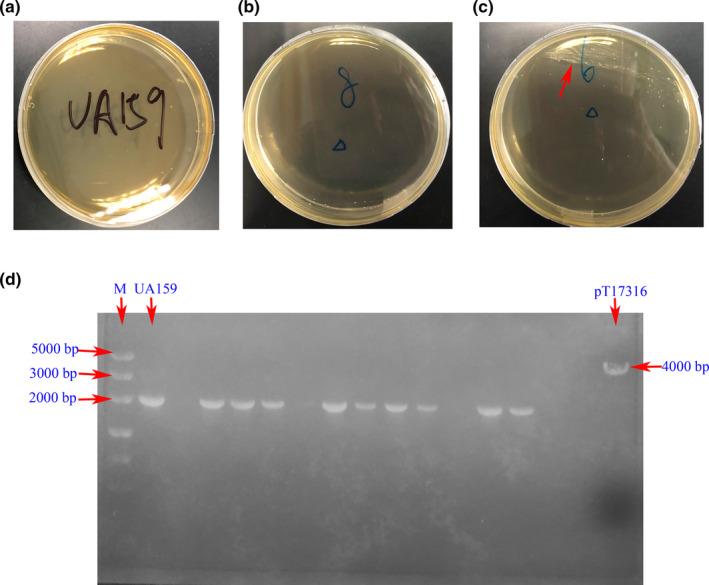
(a) Transformation plates of control culture plate of *Streptococcus mutans* UA159 with the addition of antibiotics for the sRNA0426 deletion attempt in *S. mutans*. (b) One of the representative transformation plates in which possible deletion strains were almost not grown. (c) One of the transformation plates in which few possible deletion strains were grown. (d) Representative gel electrophoresis results of the grown colony PCR. Lane M showed the DNA Ladder. The AGE lane of wild type (UA159) was shown as negative control, and the AGE lane of pT17316 was shown as a positive control. The lane of the wild type was located at 2000 bp, while the positive colony should be located at 4000 bp

In general, sRNAs regulate gene expression by base‐pairing with target mRNAs or by binding proteins directly (Chambers & Sauer, 2013) Associations between sRNA0426 and target mRNAs, including GtfB, GtfC, ComE, and CcpA, were examined, and the results further supported the role of sRNA0426 in the production of EPS biomass. The positive correlation between GtfB, GtfC, CcpA, and sRNA0426 expression, together with the negative correlation between ComE and sRNA0426, suggest that sRNA0426 might be positively associated with biofilm formation in the regulation of EPS. KEGG analysis of the predicted target genes of sRNA0426 suggests that sRNA0426 is involved in diverse physiological activities through 8 pathways (*p* < 0.05), such as metabolic pathways including carbon metabolism and microbial metabolism in diverse environments, that are associated with biofilm formation. What's more, the seed sequence is necessary but insufficient (Didiano & Hobert, 2006; Lee et al., 2016). And the most stable predicted binding sites between the biofilm‐associated mRNAs and sRNA0426 are not limited in the seed sequence. Therefore, the seed sequence of sRNA0426 might serve an important role for sRNA0426, but the association between it and the function of sRNA0426 in *S*.* mutants* on biofilm formation is needed to be further verified (Fritsch, Siqueira, & Schrank, 2018). Overall, the functions of sRNAs may be more complex than once considered. The present study is a primary exploration of biofilm‐associated sRNAs in *S*.* mutans*. The identification of more potential sRNAs and function analysis of additional sRNAs are required, and especially creating mutans to further analyze the role of sRNAs in *S*.* mutans* is needed. We have tried and failed to create mutant strains. The details are described in the Appendix A and shown in Figure A2. This could point to an essential function of this sRNA or more attempts to try.

In conclusion, we first explored the expression characteristics and potential functions of sRNAs in the biofilm formation process of standard *S*.* mutans* and clinical strains. We found that sRNA0426 and its target mRNAs are dynamically involved in the synthesis of EPS and biofilm‐associated pathways. The results presented herein suggest the presence of a novel regulator in *S*.* mutans* under biofilm conditions, providing a better understanding of the mechanism of biofilm formation.

## CONFLICT OF INTEREST

None declared.

## AUTHOR CONTRIBUTIONS

**Luoping Yin:** Data curation (equal); investigation (lead); methodology (equal); writing – original draft (equal); writing – review & editing (equal). **Wenhui Zhu:** Data curation (equal); investigation (equal); methodology (equal); writing – original draft (equal); writing – review & editing (equal). **Dongru Chen:** Software (supporting); writing – original draft (supporting). **Yan Zhou:** Investigation (supporting); methodology (supporting); writing – review & editing (equal). **Huancai Lin:** Conceptualization (lead); funding acquisition (equal); Writing – original draft (equal); writing – review & editing (equal).

## ETHICS STATEMENT

The study protocol was approved by the Ethics Committee of the Guanghua School of Stomatology, Sun Yat‐sen University (ERC‐[2015]‐09). The parents of all of the participants consented to the research.

## Data Availability

All data generated or analyzed during this study are included in this published article except the data in Appendix B (The KEGG analysis of biological pathways of the other 17 sRNAs) and Appendix C (More details about the BLASTN of 103 strains for seeking seed sequence), which are available in the figshare repository at https://doi.org/10.6084/m9.figshare.12310133.

## Appendix A

### Construction of the sRNA0426 deletion

Considering the length of sRNA0426 and the situation that there was no referable study of sRNAs deletion in *Streptococcus mutans*, we decided to attempt to construct the sRNA0426 deletion by markerless mutagenesis (Geng et al., 2016; McDaniel, Mackay, Quiroz, & Chilkoti, 2010). The knockout cassette for double‐crossover homologous recombination was amplified from p15A (Selzer, Som, Itoh, & Tomizawa, 1983), named pTG17301. pTG17301 contains a counter selectable marker (phes) (Liu et al., 2020) and antibiotics marker that works in both Gram‐negative and Gram‐positive bacteria and will not lead to polar effects. Then, primers sRNA0426 LF and sRNA0426 RF were used to amplify the left and right flanks of sRNA0426 from the regions adjacent to its coding region of *S*.* mutans* UA159, generating 971‐bp and 992‐bp amplicons. The left flank region and the right flank region were ligated to pTG17301 by seamless ligation (Tolobio Ezmax one‐step cloning Kit), creating plasmid pTC17316. And the left flank region and the right flank region were also ligated together by seamless ligation to replace the knockout cassette. The gene replacement vector was transformed into *S*.* mutans UA159* in the presence of 1 μg/μl competence‐stimulating peptide. Following the allelic exchange, *S*.* mutans* UA159 with sRNA0426 deficiency was isolated on BHI and chemically defined medium (CDM) (van de Rijn & Kessler, 1980) agar plates, with or without exogenous d‐Glu (30 mM), supplemented with 100 μg/ml antibiotics and further confirmed using colony Polymerase chain reaction (PCR) and agarose gel electrophoresis (AGE).

### Construction failure of the sRNA0426 deletion

We carried many attempts to construct mutant strains, such as circular plasmid and linear plasmid transformation by chemical transformation and electrotransformation under different conditions. All the results were negative. In the control plates of *S*.* mutans UA159*, no strain was grown (Figure A2a). In the vast majority transformation plates, almost no possible deletion strain was grown (Figure A2b). And in the very few transformation plates strain, a small number of possible deletion strains were grown (Figure A2c). In confirmation of AGE, the lane of the wild type was located at 2000 bp, while the positive colony should be located at 4000 bp. Although there are some potential positive colonies in the transformation plates, the results suggested that only the bands of 2000 bp were detected, which meant the verified strains were all false positive (Figure A2d).

### Discussion

We have tried and failed to construct a sRNA0426 mutant strain for *S*.* mutans*. This could point to an essential function of sRNA0426 or more attempts to improve the experimental methods and more trials. The present results might suggest a possibility of an important role of sRNA0426, and another situation is that homologous recombination is a probabilistic event where the times of repetitions were not enough under the tough condition of the length of sRNA0426 together with its stability. We might make a further try and confirmation of the role of sRNA0426 in the future.

**TABLE A1:** The location information for 20 sRNAs

sRNA ID	Length	Begin	End	Strand	Pre‐gene	Next‐gene	Direction	Description	Sequence
>sRNA0698	49	1,975,630	1,975,678	+	SMU_2102	SMU_2104	/−/+/+/	IGR	TATATTCTTTACTTCTATTTCTGTTCTATTTTACCACAAAAACAACAGA
>sRNA0593	103	1,744,776	1,744,878	+	SMU_1846c	SMU_1847	/−/+/−/	AM	TAACAATTTCGCCAATCATTTCATTTTCCATCAAACTTGTCCTTTCTAATAAATTCATCCAAAACTGTTTTCCTTAGGCACGGGAAACGTAGGTTCCCTCAGC
>sRNA0074	32	155,985	156,016	−	SMU_153	SMU_154	/+/−/+/	IGR	AAACGGCTACTGGAATAATGTTTAATTTTACT
>sRNA0522	132	1,501,217	1,501,348	−	SMU_1573	SMU_1574c	/−/−/−/	IGR	ATTTTCCCTTCTTAAGTTTCTTTTAAGAATCCTATCTTATACTATAGTCATCCTAGCAATAGGAATCCTAAAACTTTTCTTTTTCATAAATCTCCTAAGAATCTCAGTCCATTCGGACTGGGATTTTTTTGC
>sRNA0426	129	1,177,303	1,177,431	+	SMU_1238c	SMU_1239	/−/+/−/	AM	TAATTTCTTCAAGACTTGTCATAATAACCTCTTTCTCGTTATTAATTGGATAAGACCGTTACACAAATAATATTCGTTGAACTTTCTTGTCTCGCTATTTGATCAATTCATAAATGGCTTCTGCATAAA
>sRNA0413	118	1,140,596	1,140,713	+	SMU_1197	SMU_t37	/+/+/−/	AM	ATATTAACTAATAATAAGTCCGCAAAAATCGGGTATCAAAACTACTTTTTGTAAAAGCACCGCTTTCATCTTTACCTAATCCACCTTGAGGGAATCGAACCCCCATCTCAAGAACCGG
>sRNA0650	119	1,848,993	1,849,111	+	SMU_1975c	SMU_1976c	/−/+/−/	AM	TAATTAGCATCTTTTACATCACAATAAGTGATTGAAGAACACTCTAAGTAAAACGCCACATATGATTGTCCCATAAAGAAGAATCATCAGAGTAATCAGATAGCTGAAAGCGATATGCC
>sRNA0146	95	359,440	359,534	+	SMU_379	SMU_381c	/+/+/−/	IGR	AGAGCTAGTTGCTATAATTAATAATTTACTAGAGATTGTCTAACTGAAGAGAAGTAGTGTCTAATAGATGTTCATTATTAGCGCACGGCCATTAC
>sRNA0215	147	556,,934	557,080	−	SMU_597	SMU_598	/+/−/+/	AM	AAGCTAAGCGAGTCGCTGTTTTGATACCAATACCCGGTAATTTTGTGAAGCTCTCAATAAGTTTGGCAATAGGCGTTGGGTAGAGCATTCTTTTTCCTCACTGATTCATTGGATACATCTTTTGATAAAGATTGATGATATCTCTCG
>sRNA0120	111	287,424	287,534	+	SMU_298	SMU_299c	/+/+/−/	AM	TAACAATATGAACGATTATCTTAATGACTTAAGCGTAAGCGGCAAAACTTGCTGCACCTGAAGCCCAGCTATTTACTACAACGTCTGTTAAAGCTTGTGCTGGAGTTTTTG
>sRNA0118	100	279,927	280,026	+	SMU_291	SMU_292	/+/+/+/	IGR	TAATGTTAAAAGCTTTTAAAAACAGCTTCTTAGAAATATTGATTTTGACCTGCATCTCAAAAGTTAGCCTAAAATCTAACTTTTGGGGTGTTTTTCTATG
>sRNA0379	58	1,028,561	1,028,618	+	SMU_1083c	SMU_1084	/−/+/−/	AM	TAAGTGCTTCTTCAATTTTATCCATCGTCAACCACAACCATTCTATCCTTGCCAAAAC
>sRNA0250	88	621,927	622,014	−	SMU_662	SMU_663	/+/−/+/	AM	TCTGATGGCCATTTAAGATTCGGACTAATTCTAATCCACTATATCCTGTAATACCGACAATCGAAACTTTCATACTTATTCACTTCCT
>sRNA0301	87	745,713	745,799	−	SMU_799c	SMU_800	/−/−/+/	IGR	CTAAAGGGCAATAAAATATGTGATTCCAAAGCTTCAACAGTAACCTTTAATGGGAATATAGAATTTATAGGAAACGCTTCCAAAATT
>sRNA0600	139	1,757,677	1,757,815	+	SMU_1862	SMU_1865	/+/+/+/	IGR	TAGCTTTTCAACTTTAGCAAGAATCAGTACAACAACTCCTAGCAAAGCTGTTCGCTGTATTTGTTTCGGACCTTAGTCTCTTAGAATATCGCGTAATAGCGATTTATGCCATTTTTTACTTTAAAATCAAATAGTTGGT
>sRNA0656	113	1,849,900	1,850,012	−	SMU_1977c	SMU_1978	/−/−/−/	IGR	GTAAAAGAGATTTGACATCTCTCACTAAATAGTAATTATGGGGGATAAGATATGCTATGATCATTAAAAAGATATTTAGTCAAGAATATTTCAGTACAACTTTAGTCAAATAG
>sRNA0330	25	827,355	827,379	−	SMU_875c	SMU_876	/−/−/−/	IGR	TTTATTAGAAAGGAACAGTTTTGCA
>sRNA0187	118	460,,778	460,895	−	SMU_491	SMU_493	/+/−/+/	AM	TATCACGATAACTGTACATGCGTTCCGTCAAATAACCAAAGTGTTTTTGAGAATTCTTTTGAACATCATTTGTGTACTTTTGAGTTAAAAGCGGAATTACCATTCTCCTTCTCCTTTT
>sRNA0329	38	827,341	827,378	+	SMU_875c	SMU_876	/−/+/−/	IGR	GTATCGCAAACGTTTGCAAAACTGTTCCTTTCTAATAA
>sRNA0679	53	1,922,203	1,922,255	+	SMU_2046c	SMU_2047	/−/+/−/	AM	AATCTCAAGCAAAGACTTTTTAGATTCTAGCCTACTCCTTTTTAATCTTTTTA

Note

The data are extracted from Zhu et al. (2018). AM indicates that the sRNA is located on the antisense strand to mRNA; IGR indicates that the sRNA is in the intergenic region.

## Appendix B

**Table:**

KEGG_PATHWAY analysis for sRNA0379			
Term	Genes	%	*p* ‐value
Metabolic pathways		17.3	0.000
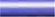
Biosynthesis of secondary metabolites		8.2	0.000
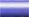
Biosynthesis of antibiotics		6	0.000
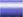
Biosynthesis of amino acids		5.2	0.000
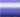
Microbial metabolism in diverse environments		4.2	0.000
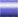
ABC transporters		3.7	0.001
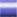
Carbon metabolism		2.8	0.010
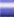
Purine metabolism		2.4	0.022
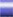
Pyrimidine metabolism		2.1	0.047
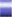

**Table:**

KEGG_PATHWAY analysis for sRNA0650			
Term	Genes	%	*p* ‐value
Metabolic pathways		17.2	0.000
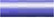
Biosynthesis of secondary metabolites		8.1	0.000
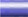
Biosynthesis of antibiotics		6	0.000
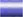
Biosynthesis of amino acids		5.2	0.000
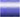
Microbial metabolism in diverse environments		4.2	0.000
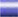
ABC transporters		3.7	0.001
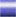
Carbon metabolism		2.8	0.011
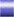
Purine metabolism		2.4	0.024
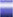
Pyrimidine metabolism		2	0.049
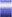

**Table:**

KEGG_PATHWAY analysis for sRNA0413			
Term	Genes	%	*p* ‐value
Metabolic pathways		17.2	0.000
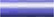
Biosynthesis of secondary metabolites		8.1	0.000
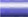
Biosynthesis of antibiotics		6	0.000
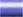
Biosynthesis of amino acids		5.2	0.000
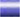
Microbial metabolism in diverse environments		4.2	0.000
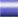
ABC transporters		3.7	0.001
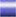
Carbon metabolism		2.8	0.012
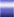
Purine metabolism		2.4	0.025
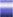

**Table:**

KEGG_PATHWAY analysis for sRNA0600			
Term	Genes	%	*p* ‐value
Metabolic pathways		17.2	0.000
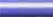
Biosynthesis of secondary metabolites		8.1	0.000
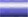
Biosynthesis of antibiotics		6	0.000
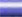
Biosynthesis of amino acids		5.2	0.000
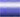
Microbial metabolism in diverse environments		4.2	0.000
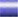
ABC transporters		3.7	0.001
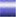
Carbon metabolism		2.8	0.012
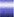
Purine metabolism		2.4	0.025
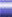

**Table:**

KEGG_PATHWAY analysis for sRNA0522			
Term	Genes	%	*p* ‐value
Metabolic pathways		17.2	0.000
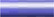
Biosynthesis of secondary metabolites		8.1	0.000
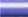
Biosynthesis of antibiotics		6	0.000
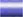
Biosynthesis of amino acids		5.2	0.000
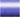
Microbial metabolism in diverse environments		4.2	0.000
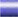
ABC transporters		3.7	0.001
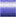
Carbon metabolism		2.8	0.011
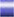
Purine metabolism		2.4	0.024
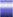
Pyrimidine metabolism		2	0.049
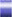

**Table:**

KEGG_PATHWAY analysis for sRNA0698			
Term	Genes	%	*p* ‐value
Metabolic pathways		17.4	0.000
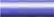
Biosynthesis of secondary metabolites		8.2	0.000
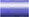
Biosynthesis of antibiotics		6	0.000
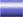
Biosynthesis of amino acids		5.2	0.000
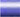
Microbial metabolism in diverse environments		4.2	0.000
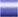
ABC transporters		3.8	0.001
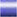
Carbon metabolism		2.8	0.009
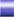
Purine metabolism		2.4	0.020
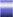
Pyrimidine metabolism		2.1	0.042
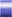

**Table:**

KEGG_PATHWAY analysis for sRNA0593			
Term	Genes	%	*p* ‐value
Metabolic pathways		17.2	0.000
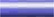
Biosynthesis of secondary metabolites		8.1	0.000
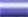
Biosynthesis of antibiotics		6	0.000
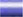
Biosynthesis of amino acids		5.2	0.000
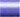
Microbial metabolism in diverse environments		4.2	0.000
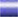
ABC transporters		3.7	0.001
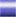
Carbon metabolism		2.8	0.012
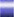
Purine metabolism		2.4	0.025
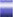

**Table:**

KEGG_PATHWAY analysis for sRNA0215			
Term	Genes	%	*p* ‐value
Metabolic pathways		17.2	0.000
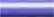
Biosynthesis of secondary metabolites		8.1	0.000
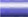
Biosynthesis of antibiotics		6	0.000
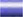
Biosynthesis of amino acids		5.2	0.000
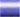
Microbial metabolism in diverse environments		4.2	0.000
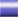
ABC transporters		3.7	0.001
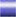
Carbon metabolism		2.8	0.012
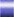
Purine metabolism		2.4	0.025
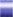

**Table:**

KEGG_PATHWAY analysis for sRNA0120			
RT	Genes	%	*p* ‐value
Metabolic pathways		17.2	0.000
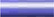
Biosynthesis of secondary metabolites		8.1	0.000
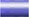
Biosynthesis of antibiotics		6	0.000
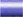
Biosynthesis of amino acids		5.2	0.000
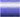
Microbial metabolism in diverse environments		4.2	0.000
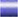
ABC transporters		3.7	0.001
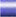
Carbon metabolism		2.8	0.012
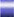
Purine metabolism		2.4	0.025
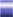

**Table:**

KEGG_PATHWAY analysis for sRNA0146			
Term	Genes	%	*p* ‐value
Metabolic pathways		17.3	0.000
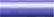
Biosynthesis of secondary metabolites		8.1	0.000
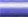
Biosynthesis of antibiotics		6	0.000
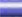
Biosynthesis of amino acids		5.2	0.000
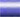
Microbial metabolism in diverse environments		4.2	0.000
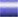
ABC transporters		3.7	0.001
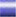
Carbon metabolism		2.8	0.011
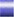
Purine metabolism		2.4	0.024
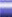
Pyrimidine metabolism		2	0.049
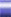

**Table:**

KEGG_PATHWAY analysis for sRNA0118			
Term	Genes	%	*p* ‐value
Metabolic pathways		17.2	0.000
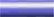
Biosynthesis of secondary metabolites		8.1	0.000
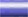
Biosynthesis of antibiotics		6	0.000
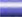
Biosynthesis of amino acids		5.2	0.000
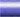
Microbial metabolism in diverse environments		4.2	0.000
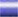
ABC transporters		3.7	0.001
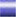
Carbon metabolism		2.8	0.012
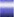
Purine metabolism		2.4	0.025
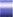

**Table:**

KEGG_PATHWAY analysis for sRNA0301			
Term	Genes	%	*p* ‐value
Metabolic pathways		17.2	0.000
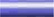
Biosynthesis of secondary metabolites		8.1	0.000
Biosynthesis of antibiotics		6	0.000
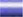
Biosynthesis of amino acids		5.2	0.000
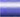
Microbial metabolism in diverse environments		4.2	0.000
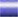
ABC transporters		3.7	0.001
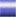
Carbon metabolism		2.8	0.012
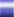
Purine metabolism		2.4	0.025
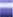

**Table:**

KEGG_PATHWAY analysis for sRNA0074			
Term	Genes	%	*p* ‐value
Metabolic pathways		17.2	0.000
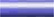
Biosynthesis of secondary metabolites		8.1	0.000
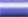
Biosynthesis of antibiotics		6	0.000
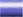
Biosynthesis of amino acids		5.2	0.000
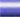
Microbial metabolism in diverse environments		4.2	0.000
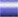
ABC transporters		3.7	0.001
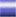
Carbon metabolism		2.8	0.012
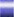
Purine metabolism		2.4	0.025
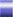

**Table:**

KEGG_PATHWAY analysis for sRNA0329			
Term	Genes	%	*p* ‐value
Metabolic pathways		17.4	0.000
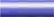
Biosynthesis of secondary metabolites		8.2	0.000
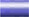
Biosynthesis of antibiotics		6	0.000
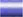
Biosynthesis of amino acids		5.2	0.000
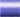
Microbial metabolism in diverse environments		4.2	0.000
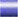
ABC transporters		3.8	0.001
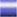
Carbon metabolism		2.8	0.010
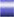
Purine metabolism		2.4	0.022
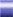
Pyrimidine metabolism		2.1	0.047
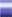

**Table:**

KEGG_PATHWAY analysis for sRNA0187			
Term	Genes	%	*p* ‐value
Metabolic pathways		17.2	0.000
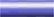
Biosynthesis of secondary metabolites		8.1	0.000
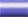
Biosynthesis of antibiotics		6	0.000
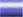
Biosynthesis of amino acids		5.2	0.000
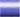
Microbial metabolism in diverse environments		4.2	0.000
ABC transporters		3.7	0.001
Carbon metabolism		2.8	0.012
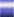
Purine metabolism		2.4	0.025
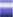

**Table:**

KEGG_PATHWAY analysis for sRNA0330			
Term	Genes	%	*p* ‐value
Metabolic pathways		17.3	0.000
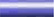
Biosynthesis of secondary metabolites		8.2	0.000
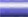
Biosynthesis of antibiotics		6	0.000
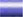
Biosynthesis of amino acids		5.2	0.000
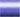
Microbial metabolism in diverse environments		4.2	0.000
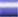
ABC transporters		3.7	0.001
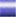
Carbon metabolism		2.8	0.012
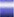
Purine metabolism		2.4	0.025
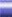

**Table:**

KEGG_PATHWAY analysis for sRNA0679			
Term	Genes	%	*p* ‐value
Metabolic pathways		17.2	0.000
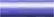
Biosynthesis of secondary metabolites		8.1	0.000
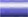
Biosynthesis of antibiotics		6	0.000
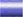
Biosynthesis of amino acids		5.2	0.000
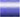
Microbial metabolism in diverse environments		4.2	0.000
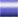
ABC transporters		3.7	0.001
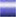
Carbon metabolism		2.8	0.010
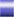
Purine metabolism		2.4	0.022
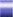
Pyrimidine metabolism		2	0.047
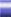

## Appendix C

Sequences producing significant alignments:

**Table:**

Description	Max score	Total score	Alignment length	Query cover	Mismatch	*E*‐value	Per.Ident	Accession
*Streptococcus mutans* strain P1	233	233	129	100%	0	0.000	100	CP050273.1
*Streptococcus mutans* strain P6	233	233	129	100%	0	0.000	100	CP050272.1
*Streptococcus mutans* strain S1	233	233	129	100%	0	0.000	100	CP050271.1
*Streptococcus mutans* strain S4	233	233	129	100%	0	0.000	100	CP050270.1
*Streptococcus mutans* strain NCH105	233	233	129	100%	0	0.000	100	CP044221.1
*Streptococcus mutans* strain UA140	233	233	129	100%	0	0.000	100	CP044495.1
*Streptococcus mutans* strain T8	233	233	129	100%	0	0.000	100	CP044492.1
*Streptococcus mutans* strain MD	233	233	129	100%	0	0.000	100	CP044493.1
*Streptococcus mutans* NBRC 13955	233	233	129	100%	0	0.000	100	AP019720.1
*Streptococcus mutans* strain NCTC10832	233	233	129	100%	0	0.000	100	LR134320.1
*Streptococcus mutans* strain NCTC10449	233	233	129	100%	0	0.000	100	LS483349.1
*Streptococcus mutans* strain LAR01	233	233	129	100%	0	0.000	100	CP023477.1
*Streptococcus mutans* strain KCOM 1054 (= ChDC YM3)	233	233	129	100%	0	0.000	100	CP021318.1
*Streptococcus mutans* strain NG8	233	233	129	100%	0	0.000	100	CP013237.1
*Streptococcus mutans* UA159‐FR	233	233	129	100%	0	0.000	100	CP007016.1
*Streptococcus mutans* GS‐5	233	233	129	100%	0	0.000	100	CP003686.1
*Streptococcus mutans* LJ23	233	233	129	100%	0	0.000	100	AP012336.1
*Streptococcus mutans* UA159	233	233	129	100%	0	0.000	100	AE014133.2
*Streptococcus mutans* NN2025	233	233	129	100%	0	0.000	100	AP010655.1
*Streptococcus mutans* strain LAB761	224	224	129	100%	2	0.000	98.45	CP033199.1
*Streptococcus troglodytae* TKU31 DNA	206	206	129	100%	6	0.000	95.35	AP014612.1
*Streptococcus oralis* subsp. *dentisani* strain F0392	58.1	58.1	34	26%	1	0.000	97.06	CP034442.1
*Streptococcus oralis* Uo5	58.1	58.1	34	26%	1	0.000	97.06	FR720602.1
*Streptococcus suis* strain WUSS351	55.4	55.4	35	27%	2	0.001	94.29	CP039462.1
*Streptococcus* sp. 1643	54.5	54.5	32	25%	1	0.002	96.88	CP040231.1
*Streptococcus* sp. oral taxon 064 strain W10853	54.5	54.5	32	25%	1	0.002	96.88	CP016207.1
*Streptococcus milleri* strain NCTC10708	53.6	53.6	34	26%	2	0.002	94.12	LR134307.1
*Streptococcus suis* strain HA1003	53.6	53.6	34	26%	2	0.002	94.12	CP030125.1
*Streptococcus suis* strain 1081	53.6	53.6	34	26%	2	0.002	94.12	CP017667.1
*Streptococcus suis* strain 0061	53.6	53.6	34	26%	2	0.002	94.12	CP017666.1
*Streptococcus pantholopis* strain TA 26	53.6	53.6	34	26%	2	0.002	94.12	CP014699.1
*Streptococcus constellatus* subsp. *pharyngis* C1050	53.6	53.6	34	26%	2	0.002	94.12	CP003859.1
*Streptococcus constellatus* subsp. *pharyngis* C818	53.6	53.6	34	26%	2	0.002	94.12	CP003840.1
*Streptococcus constellatus* subsp. *pharyngis* C232	53.6	53.6	34	26%	2	0.002	94.12	CP003800.1
*Streptococcus suis* isolate GD‐0088	50.9	50.9	35	27%	3	0.023	91.43	LR738723.1
*Streptococcus suis* isolate 861160	50.9	50.9	35	27%	3	0.023	91.43	LR738722.1
*Streptococcus suis* isolate GD‐0001	50.9	50.9	35	27%	3	0.023	91.43	LR738720.1
*Streptococcus suis* strain AH681	50.9	50.9	35	27%	3	0.023	91.43	CP025043.1
*Streptococcus* sp. 116‐D4	50	50	32	25%	2	0.023	93.75	AP021887.1
*Streptococcus mitis* strain SK637	50	50	32	25%	2	0.023	93.75	CP028415.1
*Streptococcus pneumoniae* strain 2245STDY6179186	50	50	32	25%	2	0.023	93.75	LR216066.1
*Streptococcus oralis* strain NCTC11427	50	50	32	25%	2	0.023	93.75	LR134336.1
*Streptococcus oralis* strain FDAARGOS_367	50	50	32	25%	2	0.023	93.75	CP023507.1
*Streptococcus oralis* strain S.MIT/ORALIS‐351	50	50	32	25%	2	0.023	93.75	CP019562.1
*Streptococcus mitis* strain SVGS_061	50	50	32	25%	2	0.023	93.75	CP014326.1
*Streptococcus* sp. VT 162	50	50	32	25%	2	0.023	93.75	CP007628.2
*Streptococcus pseudopneumoniae* IS7493	50	50	32	25%	2	0.023	93.75	CP002925.1
*Streptococcus pneumoniae* strain PZ900701590	49.1	49.1	34	26%	3	0.079	91.18	CP050175.1
*Streptococcus thermophilus* strain ATCC 19258	49.1	49.1	34	26%	3	0.079	91.18	CP038020.1
*Streptococcus equi* subsp. *zooepidemicus* strain TN‐714097	49.1	49.1	34	26%	3	0.079	91.18	CP046042.2
*Streptococcus equi* subsp. *zooepidemicus* strain OH‐71905	49.1	49.1	34	26%	3	0.079	91.18	CP046040.1
*Streptococcus pneumoniae* strain R6CIB17	49.1	49.1	34	26%	3	0.079	91.18	CP038808.1
*Streptococcus pneumoniae* strain 4559	49.1	49.1	34	26%	3	0.079	91.18	LR595848.1
*Streptococcus equi* subsp. *zooepidemicus* strain NCTC11854	49.1	49.1	34	26%	3	0.079	91.18	LR590471.1
*Streptococcus pneumoniae* isolate GPS_ZA_821‐sc‐1950967	49.1	49.1	34	26%	3	0.079	91.18	LR536845.1
*Streptococcus pneumoniae* strain 2245STDY6178854	49.1	49.1	34	26%	3	0.079	91.18	LR536841.1
*Streptococcus pneumoniae* strain 2245STDY6105855	49.1	49.1	34	26%	3	0.079	91.18	LR536839.1
*Streptococcus pneumoniae* strain 2245STDY6106635	49.1	49.1	34	26%	3	0.079	91.18	LR536837.1
*Streptococcus pneumoniae* strain 2245STDY6020240	49.1	49.1	34	26%	3	0.079	91.18	LR536835.1
*Streptococcus pneumoniae* strain 2245STDY5775553	49.1	49.1	34	26%	3	0.079	91.18	LR536833.1
*Streptococcus pneumoniae* strain 2245STDY5699475	49.1	49.1	34	26%	3	0.079	91.18	LR536831.1
*Streptococcus pneumoniae* strain 521	49.1	49.1	34	26%	3	0.079	91.18	CP036529.1
*Streptococcus pneumoniae* strain EF3030	49.1	49.1	34	26%	3	0.079	91.18	CP035897.1
*Streptococcus pneumoniae* strain 2245STDY6178828	49.1	49.1	34	26%	3	0.079	91.18	LR216069.1
*Streptococcus pneumoniae* isolate b04a6400‐1f66‐11e7‐b93e‐3c4a9275d6c8	49.1	49.1	34	26%	3	0.079	91.18	LR536843.1
*Streptococcus pneumoniae* isolate 55896440‐41bd‐11e5‐998e‐3c4a9275d6c6	49.1	49.1	34	26%	3	0.079	91.18	LR216065.1
*Streptococcus pneumoniae* isolate 569492b0‐41bd‐11e5‐998e‐3c4a9275d6c6	49.1	49.1	34	26%	3	0.079	91.18	LR216064.1
*Streptococcus pneumoniae* strain 2245STDY6105839	49.1	49.1	34	26%	3	0.079	91.18	LR216063.1
*Streptococcus pneumoniae* isolate GPS_HK_150‐sc‐2296816	49.1	49.1	34	26%	3	0.079	91.18	LR216062.1
*Streptococcus pneumoniae* strain 2245STDY6178826	49.1	49.1	34	26%	3	0.079	91.18	LR216061.1
*Streptococcus pneumoniae* strain 2245STDY6178787	49.1	49.1	34	26%	3	0.079	91.18	LR216060.1
*Streptococcus pneumoniae* strain 2245STDY6106384	49.1	49.1	34	26%	3	0.079	91.18	LR216057.1
*Streptococcus pneumoniae* strain 2245STDY6092949	49.1	49.1	34	26%	3	0.079	91.18	LR216055.1
*Streptococcus pneumoniae* strain 2245STDY6106372	49.1	49.1	34	26%	3	0.079	91.18	LR216054.1
*Streptococcus pneumoniae* strain 2245STDY6106337	49.1	49.1	34	26%	3	0.079	91.18	LR216051.1
*Streptococcus pneumoniae* strain 2245STDY6092834	49.1	49.1	34	26%	3	0.079	91.18	LR216048.1
*Streptococcus pneumoniae* strain 2245STDY6031034	49.1	49.1	34	26%	3	0.079	91.18	LR216047.1
*Streptococcus pneumoniae* strain 2245STDY6092613	49.1	49.1	34	26%	3	0.079	91.18	LR216046.1
*Streptococcus pneumoniae* strain 2245STDY6020221	49.1	49.1	34	26%	3	0.079	91.18	LR216045.1
*Streptococcus pneumoniae* strain 2245STDY6020210	49.1	49.1	34	26%	3	0.079	91.18	LR216043.1
*Streptococcus pneumoniae* strain 2245STDY6092581	49.1	49.1	34	26%	3	0.079	91.18	LR216042.1
*Streptococcus pneumoniae* strain 2245STDY6031048	49.1	49.1	34	26%	3	0.079	91.18	LR216041.1
*Streptococcus pneumoniae* strain 2245STDY6030848	49.1	49.1	34	26%	3	0.079	91.18	LR216040.1
*Streptococcus pneumoniae* strain 2245STDY5775610	49.1	49.1	34	26%	3	0.079	91.18	LR216039.1
*Streptococcus pneumoniae* strain 2245STDY5775666	49.1	49.1	34	26%	3	0.079	91.18	LR216037.1
*Streptococcus pneumoniae* strain 2245STDY5775603	49.1	49.1	34	26%	3	0.079	91.18	LR216036.1
*Streptococcus pneumoniae* isolate SA_GPS_SP505‐sc‐1895675	49.1	49.1	34	26%	3	0.079	91.18	LR216035.1
*Streptococcus pneumoniae* strain 2245STDY5983173	49.1	49.1	34	26%	3	0.079	91.18	LR216034.1
*Streptococcus pneumoniae* strain 2245STDY5868782	49.1	49.1	34	26%	3	0.079	91.18	LR216033.1
*Streptococcus pneumoniae* strain 2245STDY5775874	49.1	49.1	34	26%	3	0.079	91.18	LR216032.1
*Streptococcus pneumoniae* strain 2245STDY5982722	49.1	49.1	34	26%	3	0.079	91.18	LR216031.1
*Streptococcus pneumoniae* strain 2245STDY5775545	49.1	49.1	34	26%	3	0.079	91.18	LR216030.1
*Streptococcus pneumoniae* strain 2245STDY6093044	49.1	49.1	34	26%	3	0.079	91.18	LR216049.1
*Streptococcus pneumoniae* strain 2245STDY5562412	49.1	49.1	34	26%	3	0.079	91.18	LR216028.1
*Streptococcus pneumoniae* strain 2245STDY5775520	49.1	49.1	34	26%	3	0.079	91.18	LR216027.1
*Streptococcus pneumoniae* strain 2245STDY5775485	49.1	49.1	34	26%	3	0.079	91.18	LR216026.1
*Streptococcus pneumoniae* strain 2245STDY5562562	49.1	49.1	34	26%	3	0.079	91.18	LR216025.1
*Streptococcus pneumoniae* strain 2245STDY5699394	49.1	49.1	34	26%	3	0.079	91.18	LR216024.1
*Streptococcus pneumoniae* strain 2245STDY5699131	49.1	49.1	34	26%	3	0.079	91.18	LR216022.1
*Streptococcus pneumoniae* strain 2245STDY5562600	49.1	49.1	34	26%	3	0.079	91.18	LR216021.1
*Streptococcus pneumoniae* strain 2245STDY5562351	49.1	49.1	34	26%	3	0.079	91.18	LR216020.1
*Streptococcus pneumoniae* strain 2245STDY5609237	49.1	49.1	34	26%	3	0.079	91.18	LR216019.1
*Streptococcus pneumoniae* strain 2245STDY5605682	49.1	49.1	34	26%	3	0.079	91.18	LR216018.1
*Streptococcus pneumoniae* strain 2245STDY5605669	49.1	49.1	34	26%	3	0.079	91.18	LR216017.1
*Streptococcus pneumoniae* strain 2245STDY5605535	49.1	49.1	34	26%	3	0.079	91.18	LR216016.1
